# Mortality and Economic Burden of Prostate Cancer in Bulgaria: Years of Life Lost, Working Years of Life Lost, and Indirect Costs (2008–2023)

**DOI:** 10.3390/epidemiologia7010016

**Published:** 2026-01-22

**Authors:** Nadia Veleva, Konstantin Ivanov, Antonia Yaneva, Hristina Lebanova

**Affiliations:** Department of Pharmaceutical Sciences and Social Pharmacy, Faculty of Pharmacy, Medical University—Pleven, 5800 Pleven, Bulgaria; nadia.veleva@mu-pleven.bg (N.V.); konstantinworkmail@gmail.com (K.I.); antoniya.yaneva@mu-pleven.bg (A.Y.)

**Keywords:** prostate cancer, mortality, YLL, YWLL, GDP loss, indirect costs, Bulgaria, human capital approach

## Abstract

Background/Objectives: Prostate cancer is the second most common cause of cancer-related mortality among the male population worldwide. It is among the leading reasons for the increasing number of years of life lost, working years of life lost, and gross domestic product (GDP) loss in Bulgaria. The primary objective of this study is to evaluate the burden of prostate cancer in Bulgaria, including calculating years of life lost (YLL), years of working life lost (YWLL), and the associated indirect costs. Methods: An observational time-series study was conducted using official national data from the National Statistical Institute (NSI), the INFOSTAT database, and the National Social Security Institute. The study covered the period 2008–2023 and included all registered male deaths attributed to malignant neoplasm of the prostate (ICD-10: C61). YLL, YWLL, and indirect costs were calculated using the human capital approach. Due to restricted access to age-specific mortality files, additional mortality records were obtained through formal data requests to NSI. Results: Prostate cancer led to 127,457 YLL and 6345 YWLL, with productivity losses reaching €88.2 million. Mortality showed an overall increasing trend up to 2020, while YWLL declined due to deaths shifting to older age groups. Conclusions: Despite the advancements in prostate cancer diagnosis and treatment, our findings demonstrate a negative trend regarding YLL, YWLL, and indirect costs associated with the disease, in contrast to other European countries. Strengthening early screening, reducing diagnostic delays, and improving national cancer registry capacity are critical to mitigating future health and economic losses.

## 1. Introduction

Prostate cancer (PCa) is the second most common cause of cancer-related mortality among the male population worldwide, representing a significant public health challenge with substantial variations in incidence and mortality across regions [1,2,3]. While well-established risk factors include family history, hereditary syndromes, and race, differences in socioeconomic conditions and healthcare system performance also contribute to international disparities in disease burden [4,5,6].

In the European Union (EU), PCa accounts for a large proportion of cancer incidence and mortality in men, but outcomes vary considerably across member states. Western and Northern European countries report higher incidence rates, primarily due to more widespread screening and diagnostic activity, but also demonstrate higher survival rates [1]. By contrast, Central and Eastern European countries generally exhibit a lower incidence but significantly worse survival outcomes, reflecting later diagnosis and restricted access to innovative therapies [7,8].

Bulgaria exemplifies these challenges. PCa is the most frequently diagnosed malignancy among Bulgarian men (17.8%), surpassing colorectal (16.9%) and lung cancer (16.6%) [9]. At 147.1 per 100,000, the incidence rate remains below the EU average of 153.9 per 100,000 [5]. However, survival outcomes are notably poorer. The estimated 5-year survival in Bulgaria is approximately 54–68%, compared with 84–87% across the EU [1,10]. This disparity likely reflects a combination of underdiagnosis, limited use of preventive and screening examinations, and delayed access to effective treatments. According to WHO estimates, PCa mortality in Bulgaria accounted for 1.08% of total deaths in 2022, ranking the country 109th globally [11].

Beyond clinical outcomes, PCa contributes significantly to the national burden of disease through premature mortality, working years of life lost, and productivity losses. These indicators are increasingly recognized as essential for assessing the societal and economic consequences of cancer [12,13,14,15]. However, to date, no study has systematically quantified the years of life lost (YLL), years of working life lost (YWLL), and associated indirect costs equivalent to gross domestic product (GDP) loss attributable to PCa in Bulgaria.

At the global level, PCa remains one of the most commonly diagnosed malignancies in men. According to the most recent GLOBOCAN 2022 estimates, PCa accounted for approximately 1.5 million new cases and almost 400,000 deaths worldwide, representing a substantial share of the global cancer burden [16]. Incidence rates are highest in high-income regions—Australia/New Zealand, North America, and Western Europe, while mortality rates show less variability due to wide disparities in access to early detection and treatment [17].

These global patterns highlight the importance of examining national data in a broader context. Bulgaria falls within a group of countries characterized by moderate incidence but disproportionately high mortality relative to incidence, reflecting challenges in early diagnosis and access to innovative therapies. A comparison with worldwide trends therefore provides a valuable perspective for interpreting the national burden of prostate cancer and identifying priority areas for improvement.

Given this gap, the objective of the present study was to estimate the burden of PCa in Bulgaria by calculating YLL, YWLL, and indirect costs for the period 2008–2023. By providing real-world evidence on the health and economic impact of prostate cancer, this study aims to support evidence-based cancer control strategies and policy development in Bulgaria.

## 2. Materials and Methods

The study applied a mixed-methods approach, combining the following steps: desk research, public data request, and estimations. Data collection took place between August 2022 and June 2025.

### 2.1. Desk Research

This is an observational time-series using administrative mortality data aimed at assessing the burden of PCa in Bulgaria and calculating YLL and YWLL and the associated indirect costs from a societal perspective. Population, mortality, and gender-specific life expectancy data were obtained from the National Statistical Institute (NSI) for the period January 2008 to December 2023. Years of life lost were estimated based on the country- and gender-specific life expectancy. The analysis only considered the male population, given the specifics of the disease. The human capital approach was used to evaluate the indirect costs. Years of working life lost were calculated based on official country- and gender-specific retirement age data derived from the National Social Security Institute [18]. GDP loss was calculated based on official country annual GDP per employed person data from the INFOSTAT database of the Bulgarian NSI [19]. The costs of productivity losses estimated in Bulgarian levs (BGN) were converted into EUR using the fixed rate of the current currency board in Bulgaria (1 EUR = 1.95583 BGN). GDP per employed person values were used as reported in nominal terms for each respective year and were not additionally adjusted for inflation. Here, we report data from 2008 to 2023 to highlight trends in prostate cancer disease burden over the past 16 years, including the years of the COVID-19 pandemic.

### 2.2. Data Collection

Data were collected between August 2022 and June 2025. Two main data sources were used for this study (Table 1): (1) INFOSTAT data and (2) NSI data.

**Table 1:** Data sources and extracted variables.

Data Source	Description	Extracted Variables	Link	Access
National Statistical Institute INFOSTAT	Population level statistical data	Mortality, Life expectancy, Working life expectancy, GDP per employed person	https://infostat.nsi.bg/infostat/pages/external/login.jsf (Accessed on 21 August 2025)	Public
	Information on deaths from prostate cancer for the period 2008–2023, individual-level data by year and age	Number of deaths ICD-10, C61 by age	https://www.nsi.bg/ (data on file) (Accessed on 21 August 2025)	Upon request
National Social Insurance Institute	Tables for the conditions for acquiring the right to a pension for insurance years and age Table 2: Conditions for acquiring the right to a retirement-age pension under Art. 68, para. 1–2 from the Social Insurance Code for the period 2018–2037 (people with a completely insured length of service)	Gender specific retirement age	Appendix A (Table A1: 2008–2018) https://www.noi.bg/wp-content/uploads/table1_2019.pdf (Accessed on 25 June 2025)	Public

All links were accessed in June 2025.

**Table 2:** Burden of prostate cancer in Bulgaria 2008–2023.

Years	YLLs	Mean YLLs per Person	YWLLs	Mean YWLLs per Person	GDP per Person (Lost Productivity) EUR	Lost GDP per Year EUR
2008	7925.02	8.9045196	474	4.051282051	9763.97	4,628,122.88
2009	6938.38	8.4614356	264	3.180722892	9979.77	2,634,658.8
2010	6816.63	8.705789	357	3.216216216	10,623.08	3,792,437.99
2011	7859.77	8.7330783	384	3.12195122	11,768.38	4,519,057.79
2012	8086.24	8.6391426	395.35	3.561711712	12,296.66	4,861,487.25
2013	8911.41	8.9651992	434.32	3.649747899	12,289.53	5,337,588.35
2014	8149.27	8.5872199	465.32	4.081754386	12,528.62	5,829,819.63
2015	7994.37	8.2671901	370.57	4.211022727	13,293.32	4,926,106.72
2016	7989.38	8.7315674	470.04	4.796326531	14,082.44	6,619,310.43
2017	8171.19	8.4851373	421	5.134146341	14,900.77	6,273,223.53
2018	8635.32	8.287254	406.92	4.11030303	15,965.26	6,496,582.67
2019	8878.17	8.0127867	453.64	4.930869565	17,420.73	7,902,740.80
2020	8691.09	7.2789698	417.75	4.590659341	17,857.05	7,459,781.76
2021	7782.69	7.4833558	386.74	4.958205128	20,477.04	7,919,288.64
2022	7412.99	7.4055844	336.08	4.541621622	13,315.57	4,475,098.27
2023	7215.13	7.4459546	308	3.756097561	14,691.46	4,524,969.96
Total	127,457.05		6344.73			88,200,275.48

The INFOSTAT database reports were derived from the NSI website. The datasets were filtered by year and ICD-10 code of the therapeutic indication to include only malignant neoplasm of the prostate (C61). The variables of interest included mortality, life expectancy, working life expectancy, and GDP per employed person.

### 2.3. Data Requests

PCa mortality data by year were derived from the NSI database. Given the restricted access, the information was requested from the NSI by the authors through a special application, following the procedure for accessing public information.

In August 2022 and on 22 October 2024, two formal public data requests were dispatched to the NSI. The requests sought patient-level information on registered deaths from malignant neoplasm of prostate (ICD-10 code C61) by year and age, providing real-world data on mortality due to prostate cancer within the Bulgarian National Healthcare System.

### 2.4. Estimations

We estimated YLL for each year and the overall YLL for the study period.

Years of life lost (YLL)=N×L

where *N* is the number of deaths per year, and *L* is the life expectancy at age of death in years, that equals the difference between the age at death in people with PCa and the remaining life expectancy for people of the same age in the general population.

We also estimated the yearly and overall YWLL.

Years of working life lost (YWLL)=N×WL

where *N* is the number of deaths per year, and WL is the working life expectancy at the age of death in years, that equals the difference between the age at death in people with PCa and the retirement age for people of the same age, according to Art. 68, para. 1–2 from the Bulgarian Social Insurance Code for the respective year. Retirement age thresholds were based on the statutory values defined in the Bulgarian Social Insurance Code for each year during 2008–2023.

Indirect costs were estimated as the product of YWLL and GDP per employed person for the respective year. The human capital approach was selected because it estimates lifetime productivity losses and is widely used in burden-of-disease studies where mortality is the primary contributor to economic burden.

### 2.5. Analysis

The data analysis was conducted using Microsoft Excel and SPSS v24 [20]. These software tools were used for data cleaning, descriptive statistics, and correlation analysis. This process involved consolidating the data files into a unified time-series database and performing relevant estimations. The results were summarized using descriptive statistics. Pearson’s correlation coefficient was used to measure the relationship between the variables age and deaths. The significance level was set at 0.05.

## 3. Results

### 3.1. Mortality

**Figure 1:**
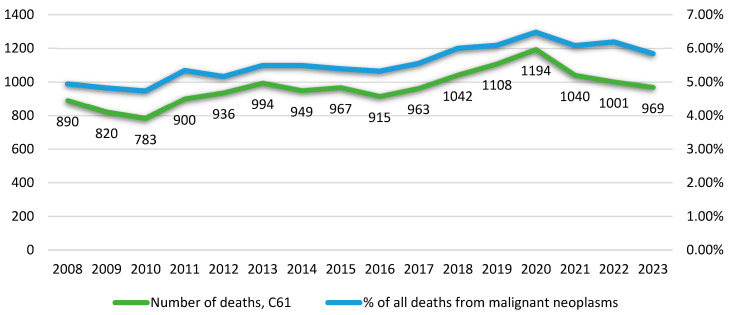
Annual number of deaths from malignant neoplasm of the prostate (ICD-10: C61) compared with total cancer mortality in Bulgaria, 2008–2023. Data source: National Statistical Institute (NSI), male population.

An estimated 15,471 deaths due to PCa occurred between 2008 and 2023 in Bulgaria (Supplementary Table S1). The mean number of deaths per year was µ = 967, SD ± 98, which accounted for an average of 5,56 per cent of all deaths from malignant neoplasm per year in Bulgaria. There is an upward trend in mortality from 2010 to 2020. The highest mortality was registered in 2020 with 1194 cases of death. While the COVID-19 pandemic could have influenced case detection and outcomes, mortality decreased by 150 cases in 2021 relative to the previous year, and this decline continued as part of a downward trend observed in 2022–2023. Prostate cancer mortality in Bulgaria follows a temporal pattern that mirrors the trend observed for all cancer-related mortality in the country (Figure 1).

**Figure 2:**
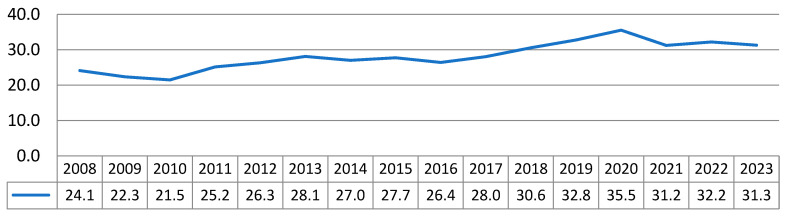
Mortality rate for malignant neoplasm of the prostate (ICD-10: C61) in Bulgaria, expressed per 100,000 male population, 2008–2023. Data source: National Statistical Institute (NSI).

The estimated mean mortality rate in Bulgaria for the study period was µ = 28.1, SD ± 3.78 per year per 100,000 population (Figure 2).

Bulgaria has a higher standardized mortality rate compared to the EU average, but no improvement in this indicator has been observed [8]. Contrary to the EU trend, the prostate cancer mortality in Bulgaria is increasing by 19% between 2011 and 2019 [21].

### 3.2. Years of Life Lost

**Figure 3:**
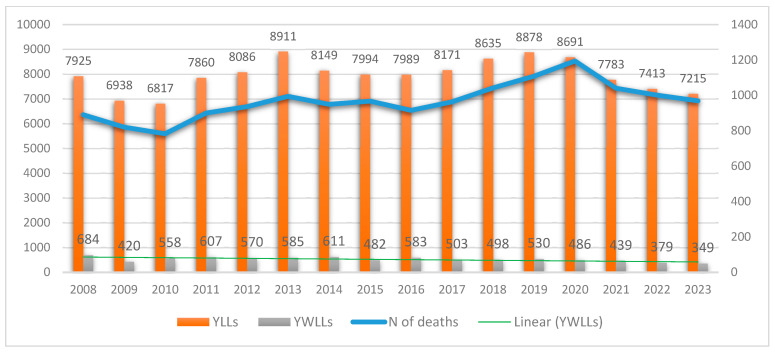
Annual distribution of prostate cancer deaths, years of life lost (YLL), and years of working life lost (YWLL) in Bulgaria between 2008 and 2023. YLL and YWLL are calculated using life expectancy and statutory retirement-age thresholds for each year. Data source: National Statistical Institute (NSI), INFOSTAT database.

A cumulative loss of approximately 127,457 life years occurred in Bulgaria between 2008 and 2023 (Figure 3). There was a downward trend in YLL during the first two years, followed by fluctuations over three years. Even though the number of YLL due to PCa has increased in Bulgaria for the entire study period, the average YLL per person remains stable, with µ = 8, SD ± 0.53, and even decreases to about 7.4 YLL per person during the last four years (Table 1).

**Figure 4:**
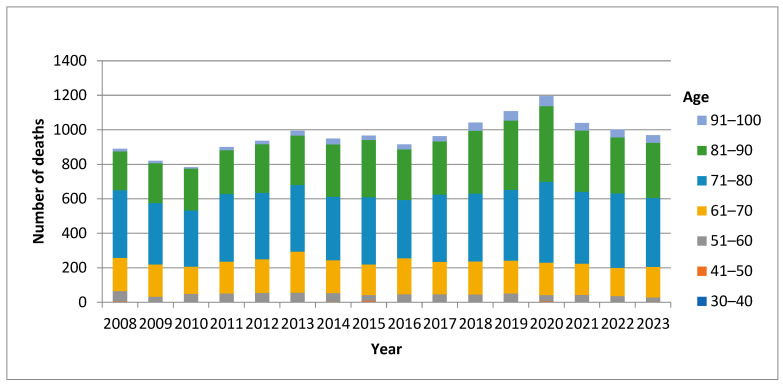
Number of deaths from malignant neoplasm of the prostate (ICD-10: C61) by age group in Bulgaria, 2008–2023. The figure illustrates the age distribution of mortality, with the highest burden observed among men aged 71–80 years. In the 30–40 age group, deaths were recorded only sporadically during the study period: one case in 2010, one in 2012, two in 2014, and one case each in 2015, 2016, 2017, and 2019. Data source: National Statistical Institute (NSI). Our results confirm those of other studies, as we found a moderate positive correlation between age and number of deaths from PCa (r = 0.457, N = 68, *p* = 0.000) [8].

Analysis of the distribution of deaths due to PCa in particular age groups (Figure 4) indicated a significant variability with age. While in the youngest age group (30–40) there were single cases of premature death, in the population aged 71–80 the burden of premature death was the highest, followed by the age groups (81–90) and (61–70).

N is lower than 100 because no death cases were registered within the range from 0 to 30 years of age, and three age groups (0–10, 11–20, and 21–30) were excluded from the analysis. It is well known that PCa mainly affects males over fifty years of age. In our dataset, PCa mortality was rarely observed in individuals younger than 40 years; a single case was documented at age 33. Accordingly, 30 years was selected as the lower bound for the age distribution in the analysis.

Population ageing impacts cancer incidence differently across countries, with estimated increases in new cancer diagnoses ranging from +2% in Latvia to +57% in Luxembourg. The effect of population ageing is more substantial on cancer mortality and varies also widely among countries, from +6% in Bulgaria to +71% in Iceland [8].

### 3.3. Years of Working Life Lost

**Figure 5:**
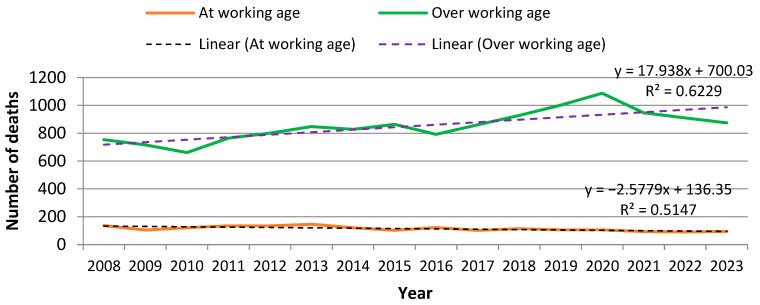
Distribution of prostate cancer deaths in Bulgaria by work status (working-age vs. post-retirement) for the period 2008–2023. The figure highlights that most deaths occurred among men older than the statutory retirement age. Data source: National Statistical Institute (NSI), with retirement thresholds defined by the Social Insurance Code.

A cumulative loss of approximately 6345 working life years occurred between 2008 and 2023. Mean YWLL per person were µ = 4, SD ± 0.64 (Table 2). While the number of deaths and the YLL due to PCa have increased over the study period, the years of working life lost have demonstrated a decreasing trend. This can be explained by the fact that the largest share of death cases was attributable to men at retirement age. The number of deaths due to PCa in the working age group in Bulgaria between 2008 and 2023 was seven times lower, with 1831 cases, than that in the group above working age, with 13,559 cases. Furthermore, a trend towards a decrease in mortality was observed in the working-age group during the entire 16-year period (R^2^ = 0.5147). This pattern reflects the shift in mortality toward older age groups who are beyond the statutory retirement age. The distribution of deaths due to prostate cancer according to work status is presented in Figure 5.

### 3.4. Indirect Costs

**Figure 6:**
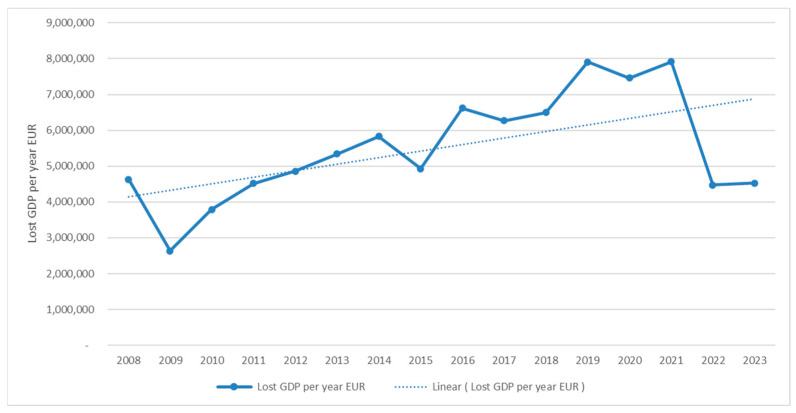
Estimated annual productivity loss (GDP-equivalent) attributable to prostate cancer in Bulgaria, 2008–2023. Indirect costs are calculated using the human-capital approach and GDP per employed person for each corresponding year. Data source: National Statistical Institute (NSI), INFOSTAT GDP statistics.

The overall indirect costs measured as productivity losses due to PCa were estimated at 88,200,277 EUR for the study period in Bulgaria. The mean annual GDP loss was 5,512,517 EUR. The mean GDP loss per person was 13,824 EUR. The most considerable GDP losses were observed between 2019 and 2021, which can be attributed to the COVID-19 pandemic leading to an increase in mortality among working-age individuals (see Figure 6). Although GDP loss is a negative indicator, it demonstrated a positive change during the study period, considering the significantly lower mortality from PCa in the working-age group and the decreasing trend in YWLL (Figure 5). Still, according to the current Bulgarian legislation (Art. 68, para. 1–2 from the Social Insurance Code for the period 2018–2037), the statutory retirement age will be increasing yearly until 2037, which would most probably cause increasing mortality and increasing GDP loss as more people from the riskier age groups will not be able to leave the workforce.

## 4. Discussion

### 4.1. Mortality Trends

PCa remains a substantial public health challenge in Bulgaria, with mortality demonstrating a modest upward trend between 2008 and 2023. The observed peak of 1198 deaths in 2020 coincided with the COVID-19 pandemic, which may have contributed to excess mortality through delayed diagnosis, reduced access to health services, and interruptions in treatment pathways, patterns reported for other cancers across Europe during the pandemic period [22,23,24]. The subsequent decline in mortality during 2021–2023 suggests partial recovery of healthcare capacity, although the long-term effects of the pandemic on prostate cancer detection and survival remain uncertain.

When compared with global estimates, Bulgaria exhibits patterns consistent with countries where PCa mortality remains high despite relatively lower incidence. Worldwide, incidence has risen steadily due to population ageing and increased detection through PSA testing, whereas mortality has stabilized or declined in many high-income countries ([16,17]. In contrast, Bulgaria shows rising mortality between 2008 and 2020, aligning more closely with global regions where limited screening and delayed access to effective treatments contribute to worse outcomes. This discrepancy reinforces the need for improved early detection strategies and accelerated uptake of contemporary therapies in Bulgaria.

### 4.2. Economic Burden

Our findings confirm that the burden of PCa in Bulgaria, measured by years of life lost (YLL) and years of working life lost (YWLL), remains considerable. Between 2008 and 2023, a cumulative loss of ~127,000 life years and 6000 working years was documented, corresponding to productivity losses exceeding €88 million. Although these values are numerically lower than in Western European countries due to Bulgaria’s smaller population size, the relative burden is proportionally higher when accounting for survival differences and health system performance [7,25]. The average YLL per patient (8 years) remained relatively stable, whereas YWLL showed a decreasing trend, reflecting the predominance of deaths among older men above retirement age.

### 4.3. Comparison with European and Global Data

The comparatively low 5-year survival rate for PCa in Bulgaria (~68%) relative to the EU average (~87%) underscores persistent disparities in early detection and access to effective treatment [2,10,26]. Importantly, this gap persists despite significant therapeutic advances during the study period, including the approval of novel androgen receptor-targeted agents such as enzalutamide, apalutamide, darolutamide, and abiraterone, which have demonstrated significant survival benefits in metastatic and advanced disease [27,28,29,30,31,32,33,34,35,36]. While these therapies were rapidly integrated into standard care across much of the European Union, their adoption and accessibility in Bulgaria may have been more limited, further contributing to survival disparities [37].

### 4.4. Policy Implications

Access to innovative therapies in Eastern Europe, including Bulgaria, has historically lagged behind Western Europe due to delays in health technology assessment, reimbursement approvals, and fiscal constraints [38,39]. Such systemic barriers may have restricted the timely uptake of new agents, diminishing their potential population-level impact. The time to reimbursement since obtaining the first marketing authorization from European medicines agency (EMA) is 594 days for abiraterone, 540 days for enzalutamide, 719 days for apalutamide and 643 days for darolutamide [40]. These access inequalities, when combined with the limited use of preventive examinations and the absence of systematic screening programs, likely contribute to later-stage diagnoses and poorer outcomes compared to those in the EU.

In line with regional projections, population ageing will further intensify the burden of PCa in Bulgaria [8]. Rising retirement ages are expected to increase the share of at-risk men remaining in the workforce, potentially elevating both mortality and productivity losses linked to premature death [11]. These projections underscore the necessity for comprehensive cancer control strategies, encompassing population-based screening initiatives, equitable access to innovative therapies, and the revitalization of the Bulgarian National Cancer Registry to ensure reliable monitoring of incidence, survival rates, and outcomes. Actionable interventions include implementing short-term measures, such as structured early-detection programmes, reducing diagnostic delays in primary care, and long-term measures, such as restoring a fully functional national cancer registry to ensure timely and accurate data collection. Such measures would help diagnose patients in earlier stages of the disease, reduce premature mortality, and align outcomes in Bulgaria with those of other EU member states.

In summary, PCa in Bulgaria imposes a substantial and growing burden, reflected in high YLL and YWLL, persistent survival disparities, and significant economic losses. Despite therapeutic progress at the global level, Bulgaria’s outcomes remain markedly worse than the EU average. Our findings emphasize the need for urgent health system interventions focused on early detection, accelerated access to novel treatments, and strengthened cancer surveillance infrastructure to reduce inequalities and alleviate the societal and economic impact of prostate cancer in Bulgaria.

## 5. Conclusions

PCa imposes a significant and persistent health and economic burden in Bulgaria. The cumulative YLL and YWLL between 2008 and 2023 reveal not only high levels of premature mortality but also considerable productivity losses, despite the availability of effective therapies at the European level. Mortality trends, coupled with comparatively low survival rates, highlight structural weaknesses in early detection, treatment access, and cancer registry infrastructure.

The introduction of novel androgen receptor-targeted therapies during the study period, including enzalutamide, apalutamide, darolutamide, and abiraterone, has improved outcomes in many European countries. However, delayed adoption and limited accessibility in Bulgaria may have diminished their potential population-level impact. Combined with the underutilization of preventive and screening services, these barriers have likely contributed to persistent survival disparities compared with EU averages.

Strengthening cancer control policies should therefore be a national priority. Key measures include developing systematic screening and prevention programs, accelerating access to innovative therapies through timely reimbursement, and re-establishing a fully functional national cancer registry. Implementation of such strategies is essential to reduce premature mortality, improve survival, and mitigate the growing social and economic burden of prostate cancer in Bulgaria.

## 6. Strengths and Limitations of the Study

A key strength of this study is the use of real-world, population-based data covering a 16-year period, which enabled the estimation of years of life lost (YLL), years of working life lost (YWLL), and indirect costs attributable to productivity costs due to prostate cancer for the first time in Bulgaria. The analysis integrates epidemiological and economic perspectives, providing a comprehensive assessment of the disease burden that may inform both clinical practice and policy development. However, several limitations must be acknowledged. First, mortality data was derived from the National Statistical Institute, and only NSI-registered prostate cancer deaths were included. The absence of a fully functional cancer registry restricts survival and stage-at-diagnosis analyses. This reliance on administrative records may have led to an underestimation of the actual burden. Second, survival data and stage at diagnosis were unavailable, limiting the ability to contextualize mortality trends in terms of treatment effectiveness and disease progression. Third, the use of the human capital approach to estimate productivity losses may overestimate the economic impact compared with alternative valuation methods, such as the friction cost approach [41]. Fourth, while international comparisons were made where possible, differences in data sources, coding practices, and health system characteristics across countries may affect the comparability of the results. Finally, due to data constraints, counterfactual modelling of expected deaths during the COVID-19 period and calculation of confidence intervals for YLL and indirect costs were not performed.

Despite these limitations, the study provides novel and robust evidence on the health and economic burden of prostate cancer in Bulgaria, filling a critical knowledge gap and offering a foundation for evidence-based cancer control strategies.

## Data Availability

Data is contained within the article or Supplementary Materials.

## Supplementary Materials

The following supporting information can be downloaded at https://www.mdpi.com/article/10.3390/epidemiologia7010016/s1, Table S1.

## Appendix A

**Table A1:** Conditions for acquiring the right to a retirement-age pension for the period 2008–2018.

Year	Women		Men	
	Age	Length of Service	Age	Length of Service
2008	59 y 6 m	34 y	63 y	37 y
2009	60 y	34 y	63 y	37 y
2010	60 y	34 y	63 y	37 y
2011	60 y	34 y	63 y	37 y
2012	60 y 4 m	34 y 4 m	63 y 4 m	37 y 4 m
2013	60 y 8 m	34 y 8 m	63 y 8 m	37 y 8 m
2014	60 y 8 m	34 y 8 m	63 y 8 m	37 y 8 m
2015	60 y 8 m	35 y	60 y 8 m	38 y
2016	60 y 10 m	35 y 2 m	63 y 10 m	38 y 2 m
2017	61 y	35 y 4 m	64 y	38 y 4 m
2018	61 y 2 m	35 y 6 m	64 y 1	38 y 6 m
