# Supplementary material for: Mortality and Economic Burden of Prostate Cancer in Bulgaria: Years of Life Lost, Working Years of Life Lost, and Indirect Costs (2008–2023)
Source: Epidemiologia (Basel). 2026 Jan 22;7(1):16. doi: 10.3390/epidemiologia7010016 (PMC12921993; doi:10.3390/epidemiologia7010016)
Supplement: Supplementary file 1 [file epidemiologia-07-00016-s001.zip › epidemiologia-3868962-supplementary.pdf]

Mortality causes (prostate cancer)		2008	2009	2010	2011	2012	2013	2014	2015
	Total	890	820	783	900	936	994	949	967
	0	0	0	0	0	0	0	0	0
	1	0	0	0	0	0	0	0	0
	2	0	0	0	0	0	0	0	0
	3	0	0	0	0	0	0	0	0
	4	0	0	0	0	0	0	0	0
	5	0	0	0	0	0	0	0	0
	6	0	0	0	0	0	0	0	0
	7	0	0	0	0	0	0	0	0
	8	0	0	0	0	0	0	0	0
	9	0	0	0	0	0	0	0	0
	10	0	0	0	0	0	0	0	0
	11	0	0	0	0	0	0	0	0
	12	0	0	0	0	0	0	0	0
	13	0	0	0	0	0	0	0	0
	14	0	0	0	0	0	0	0	0
	15	0	0	0	0	0	0	0	0
	16	0	0	0	0	0	0	0	0
	17	0	0	0	0	0	0	0	0
	18	0	0	0	0	0	0	0	0
	19	0	0	0	0	0	0	0	0
	20	0	0	0	0	0	0	0	0
	21	0	0	0	0	0	0	0	0
	22	0	0	0	0	0	0	0	0
	23	0	0	0	0	0	0	0	0
	24	0	0	0	0	0	0	0	0
	25	0	0	0	0	0	0	0	0
	26	0	0	0	0	0	0	0	0
	27	0	0	0	0	0	0	0	0
	28	0	0	0	0	0	0	0	0
	29	0	0	0	0	0	0	0	0
	30	0	0	0	0	0	0	0	0
	31	0	0	0	0	0	0	0	0

32	0	0	0	0	0	0	0	0
33	0	0	1	0	0	0	0	0
34	0	0	0	0	0	0	2	0
35	0	0	0	0	0	0	0	0
36	0	0	0	0	0	0	0	0
37	0	0	0	0	0	0	0	0
38	0	0	0	0	0	0	0	1
39	0	0	0	0	0	0	0	0
40	0	0	0	0	1	0	0	0
41	0	0	0	0	0	0	0	0
42	0	1	0	0	0	0	0	0
43	1	0	0	0	0	0	0	1
44	1	0	0	0	0	0	1	0
45	0	0	0	0	0	0	0	0
46	0	0	0	0	0	0	0	0
47	1	0	0	0	0	0	1	0
48	2	1	1	1	0	2	0	2
49	1	0	1	0	0	1	1	1
50	0	2	0	2	1	0	0	4
51	2	0	2	1	2	0	1	0
52	2	1	1	2	1	2	2	2
53	6	4	0	5	0	2	3	0
54	2	1	4	2	1	2	2	0
55	3	0	3	7	4	5	3	0
56	4	3	5	4	8	5	2	5
57	8	3	2	6	4	6	4	1
58	9	5	7	8	15	13	5	6
59	12	9	12	7	9	6	12	8
60	11	2	10	6	9	12	14	11
61	6	13	13	9	9	8	11	7
62	13	10	17	17	16	14	13	10
63	9	18	11	23	15	18	19	12
64	24	10	21	23	16	23	18	17
65	19	21	10	12	24	27	7	15
66	17	26	9	13	20	32	25	19
67	37	14	14	26	20	31	22	18
68	23	26	24	19	29	24	27	25
69	21	25	18	16	23	23	23	31
70	24	24	21	27	22	37	26	23
71	29	31	27	25	30	19	37	25
72	31	26	23	37	27	29	26	27
73	38	25	21	28	28	28	23	38
74	42	38	39	36	39	37	31	39
75	34	48	36	44	35	34	29	37

	76	46	36	29	42	45	47	44	35
	77	42	32	51	41	49	44	38	49
	78	41	41	34	46	43	61	48	42
	79	45	37	37	42	55	42	41	53
	80	44	42	28	51	35	46	51	45
	81	40	40	41	43	57	52	48	51
	82	40	31	48	37	27	33	40	44
	83	32	35	32	33	35	47	44	35
	84	30	24	34	28	36	41	43	47
	85	30	26	28	39	35	37	31	33
	86	15	17	21	24	30	23	33	34
	87	15	21	17	16	24	18	25	28
	88	14	15	8	16	20	20	17	27
	89	7	15	9	11	12	10	17	20
	90	2	7	5	7	6	6	6	13
	91	1	3	4	8	10	12	11	6
	92	2	0	1	3	6	5	5	9
	93	7	6	0	2	0	5	8	3
	94	2	5	0	2	0	4	4	3
	95	2	0	1	1	1	1	1	3
	96	0	0	1	1	1	0	1	1
	97	1	0	0	0	1	0	1	1
	98	0	0	0	1	0	0	1	0
	99	0	0	0	0	0	0	0	0
Age	100+	0	0	1	0	0	0	1	0
Total		890	820	783	900	936	994	949	967

2016	2017	2018	2019	2020	2021	2022	2023
915	963	1042	1108	1194	1040	1001	969
0	0	0	0	0	0	0	0
0	0	0	0	0	0	0	0
0	0	0	0	0	0	0	0
0	0	0	0	0	0	0	0
0	0	0	0	0	0	0	0
0	0	0	0	0	0	0	0
0	0	0	0	0	0	0	0
0	0	0	0	0	0	0	0
0	0	0	0	0	0	0	0
0	0	0	0	0	0	0	0
0	0	0	0	0	0	0	0
0	0	0	0	0	0	0	0
0	0	0	0	0	0	0	0
0	0	0	0	0	0	0	0
0	0	0	0	0	0	0	0
0	0	0	0	0	0	0	0
0	0	0	0	0	0	0	0
0	0	0	0	0	0	0	0
0	0	0	0	0	0	0	0
0	0	0	0	0	0	0	0
0	0	0	0	0	0	0	0
0	0	0	0	0	0	0	0
0	0	0	0	0	0	0	0
0	0	0	0	0	0	0	0
0	0	0	0	0	0	0	0
0	0	0	0	0	0	0	0
0	0	0	0	0	0	0	0
0	0	0	0	0	0	0	0
0	0	0	0	0	0	0	0
0	0	0	0	0	0	0	0
0	0	0	0	0	0	0	0
0	0	0	0	0	0	0	0

0	0	0	0	0	0	0	0
0	0	0	0	0	0	0	0
0	0	0	0	0	0	0	0
0	0	0	0	0	0	0	0
0	0	0	1	0	0	0	0
0	1	0	0	0	0	0	0
0	0	0	0	0	0	0	0
0	0	0	0	0	0	0	0
1	0	0	0	0	0	0	0
0	0	1	0	0	0	0	0
0	0	0	0	0	0	0	0
0	0	0	0	0	0	0	0
0	1	0	0	0	0	0	0
1	0	0	0	0	0	0	0
1	0	0	1	0	0	1	1
1	0	1	0	0	0	0	0
0	1	0	0	2	0	0	0
0	1	0	0	2	1	0	0
0	0	2	2	3	1	0	0
1	0	1	1	3	2	3	0
5	1	1	1	2	3	1	2
6	2	0	2	1	2	2	2
2	2	3	2	3	2	3	1
4	4	1	0	2	0	0	2
0	6	5	4	0	5	0	1
6	8	10	10	1	5	4	2
4	7	4	5	4	6	5	1
9	8	8	5	8	6	9	10
5	4	8	17	11	10	8	6
13	8	7	15	8	8	6	9
13	10	14	9	10	7	9	17
16	7	12	9	10	9	13	12
10	11	21	8	21	11	10	16
25	20	15	14	15	15	17	13
21	25	20	19	18	14	20	13
21	25	24	23	23	29	12	24
30	27	19	35	21	27	17	29
31	27	30	27	33	26	25	18
29	28	30	31	29	35	34	26
21	28	31	27	47	41	29	27
24	33	44	38	40	31	48	44
32	28	35	40	49	42	40	45
36	36	30	40	45	49	40	40
32	38	55	48	31	38	52	35

41	46	33	44	45	38	54	37
40	31	36	48	75	41	49	49
29	48	50	45	44	43	42	40
34	46	31	42	50	44	39	41
49	56	48	39	43	50	39	42
41	39	42	38	56	47	44	52
41	49	47	55	49	46	48	51
41	41	50	47	49	42	30	25
32	32	48	46	50	30	37	33
32	40	40	56	42	40	37	34
31	34	42	44	49	41	28	25
22	29	35	40	49	33	32	31
21	15	17	33	41	35	19	24
18	13	29	25	30	17	28	23
15	17	14	18	23	24	22	22
10	14	17	23	19	14	9	19
7	5	8	10	12	13	11	8
5	3	9	9	4	6	6	8
2	3	5	2	6	6	7	4
2	4	4	3	6	2	7	2
2	0	2	3	5	1	1	1
0	1	3	1	2	1	2	2
0	0	0	2	1	0	2	0
0	0	0	1	1	0	0	0
0	0	0	0	1	1	0	0
915	963	1042	1108	1194	1040	1001	969
